# Regulatory science - JEMS symposium in 2014

**DOI:** 10.1186/s41021-015-0012-x

**Published:** 2015-07-30

**Authors:** Hajime Kojima, Toshio Kasamatsu

**Affiliations:** Japanese Center for the Validation of Alternative Methods, Division of Pharmacology, Biological Safety Research Center, National Institute of Health Sciences, Tokyo, Japan; R&D - Safety Science Research, Kao Corporation, 2606 Akabane, Ichikai-Machi, Haga-Gun, Tochigi 321-3497 Japan

**Keywords:** Regulatory science, Food safety commission of Japan, Risk assessment, Risk management

## Abstract

The Public Symposium of the Japanese Environmental Mutagen Society (JEMS), entitled “Regulatory Science”, was held at the Shiba-Kyoritsu campus of Keio University, Tokyo, on May 24, 2014. The concept of regulatory science was first proposed by Dr. Mitsuru Uchiyama in 1987 as the science that reconciles the fruits of science and technology with the most desirable form for harmony between people and society, by making accurate predictions, assessments, and judgments, based on evidence. However, a quarter-century later, the perception (or attitude) of the masses towards this word appears to be diverse. The main aim of this symposium was to provide the concerned population, including the members of JEMS, with an opportunity to rediscover the role played by JEMS in regulatory science and to discuss the meaning and implications of regulatory science, as well as its development in Japan. The morning session comprised six lectures, including one special lecture, which introduced the regulatory science-related activities of the organizations closely relevant to JEMS. The afternoon session included five lectures on the issues of regulatory science, pertinent to the Food Safety Commission of Japan (FSCJ), which were followed by a panel discussion. This symposium highlighted the important contributions of JEMS toward development of regulatory science in Japan, in conjunction with the many challenges to be addressed. We believe that this symposium provided great scope for eliciting interest in regulatory science, in many members of the audience. It would be helpful if all members of the audience, as well as the readers of this article, would attempt to clarify the challenges presented in this symposium.

**Electronic supplementary material:**

The online version of this article (doi:10.1186/s41021-015-0012-x) contains supplementary material, which is available to authorized users.

## Introduction

The term “regulatory science” is getting popular in fields of regulation of chemicals recently. The concept of regulatory science was initially proposed by Dr. Mitsuru Uchiyama in 1987 as the science that reconciles the fruits of science and technology with the most desirable form of harmony between people and society, by making accurate predictions, assessments, and judgments, based on evidence. However, a quarter-century later, the perception or attitude of the masses towards this word appears to be diverse. JEMS was established approximately 40 years ago, in order to adapt to the urgent need for the identification and evaluation of environmental mutagens during a period of high growth in Japan. Since then, JEMS has made an important contribution towards the identification and evaluation of mutagens as well as the establishment of testing guidelines for genotoxicity evaluation.

We wish to provide the concerned population, including the members of JEMS, with an opportunity to rediscover the role played by JEMS in the development and propagation of regulatory science, and to discuss the meaning and implications of regulatory science, as well as its development in Japan. Following this, experts active in the organizations associated with the regulation of environmental mutagens were requested to introduce the latest proceedings in regulatory science. Furthermore, experts involved in risk assessment, mainly in the Food Safety Commission of Japan (FSCJ), were requested to present a lecture based on their experience, regarding challenges toward the development of regulatory science in Japan. Past discussions regarding regulatory science have mostly focused on approaches toward addressing the regulation of medical drugs, Therefore, in this symposium, we attempted to obtain novel insights into this discussion, from another area of concern. Food is a complex substance composed of various chemicals (nutrients) with a large level of exposure, i.e., large quantities of food chemicals are ingested compared to chemicals comprising medical/pharmaceutical products. In addition, food is ingested by a large proportion of the population, whereas only a small subset of these intake drugs/pharmaceuticals. Therefore, different approaches and/or challenges must be applied to assure food safety, in the context of regulatory science. Accordingly, a panel discussion was held with the lecturers, in order to improve their understanding of the challenges faced by the regulatory science organizations in Japan. This was accomplished via the exchange of views and ideas with the audience.

### Summary of the presentations

Eleven experts gave lectures on the various aspects of regulatory science in this symposium. This section provides summaries of the lectures presented by nine experts. The contents of the lectures by Drs. Takei and Kanno are provided as short reviews in the same volume of Genes and Environment [1, 2].

### “Practice of regulatory science at the National Institute of Health Sciences” by Dr. Tohru Kawanishi, National Institute of Health Sciences (NIHS)

Over a quarter of a century has passed since the proposition by Dr. Mitsuru Uchiyama, the former Director-General of the NIHS, that regulatory science is the science that reconciles the fruits of science and technology with the most desirable form for harmony between people and society, by making accurate predictions, assessments, and judgments, based on evidence [3]. During this time, regulatory science has been utilized to form the basis for research concerning the evaluation of quality, efficacy, and safety of pharmaceuticals, biologicals, medical devices, regenerating products, etc., in the field of evaluation of medical products. In addition, regulatory science assisted in the establishment of guidelines for the development and review of these products. In the field of food safety, regulatory science has been defined as the science that supports risk analysis, which is used to develop an estimate of the risks to human health and safety, identify and implement appropriate measures for the control of these risks, and communicate with stakeholders regarding the risks and the applied preventive measures. Moreover, the promotion of regulatory science has been highlighted in the Japanese policy papers in recent years. Dr. Kawanishi summarizes the rationale and background behind RS development. In addition, the regulatory science-related activities of NIHS have been introduced.

Advanced medical technologies must be developed for the establishment of an ultra-aging society in Japan, where people can enjoy long and healthy lives. In order to facilitate this, a national policy for research, development, and promotion of medical products, and the improvement of research and development (R&D) environments for R&D is currently being formulated. In Japan, many ideas for the development of pharmaceuticals have originated from academia including universities. However, almost all of the cutting-edge medical products are first commercialized in Europe and the United States, resulting in a so-called drug lag and device lag in Japan. One of the major reasons being highlighted for this is the insufficiency in the regulatory system, including the application for and processing of government approvals. In order to overcome this problem, the “Japan Revitalization Strategy (June 2013)” emphasizes the enhancement and promotion of regulatory science, which supports the R&D of medical products of Japanese origin. In addition, Act on promotion of healthcare policy, which would be enforced in the autumn of 2014, includes Article 13–2: In order to promote science (= regulatory science) touching the appropriate and rapid predicts, assessments, and judgments of their quality, efficacy and safety on the basis of scientific evidence on the occasion of the practical use of medical products, the state shall (a) establish necessary organizations; (b) secure and train necessary human resource, and improve their quality; and (c) take other measures.

Currently, the NIHS is focusing on three priority subjects: 1) regulatory science research, supporting the R&D of cutting-edge medical products, 2) regulatory science research in order to ensure the safety of food and chemicals in living environment, and 3) regulatory science that enhances the ability to perform the required tests and investigations in Japanese laboratories. According to the policy described above, the divisions in the NIHS related to medical products have initiated regulatory science research in 2012 to improve the developing environment of innovative medical products. These innovative medical products include nano-medicines, fully engineered protein drugs, nucleic acid drugs, gene-therapy drugs, cell-therapy products, and tissue-engineered medical products. The current focus of the regulatory science research is the development of point-to-consider documents to evaluate the quality and non-clinical safety of these products. These documents will also summarize the conditions for the first in-human trial, especially from the quality and non-clinical safety evaluation standpoints. The standard methods for the evaluation of quality, efficacy, and safety of these products have also been developed.

### “Global trends in regulatory safety assessment” by Dr. Hajime Kojima, Japanese Center for the Validation of Alternative Methods, Division of Pharmacology, Biological Safety Research Center, NIHS

Recent international projects concerning regulatory safety assessment of chemicals include Tox21 [4], ToxCast™ [5], SEURAT-1 [6], and ARCH-Tox [7]. Overviews of these projects were presented at the 9th World Congress on Alternatives and Animal Use in the Life Sciences (WC9) in 2014 [8], and each is expected to have an impact on future safety assessment of chemicals.

In USA, Tox21 pools federal resources and expertise from the Environmental Protection Agency (EPA), National Institutes of Environmental Health Sciences/National Toxicology Program, National Institutes of Health, National Center for Advancing Translational Sciences, and the Food and Drug Administration in a program that utilizes robotics technology to screen thousands of chemicals for potential toxicity, using screening data to predict the potential toxicity of chemicals and developing a cost-effective approach for prioritizing the thousands of chemicals that need toxicity testing. The Tox21 consortium leverages its partners’ resources and expertise to predict more effectively how a collection of 10,000 compounds comprising environmental chemicals and approved drugs will affect human health and the environment.

A major part of the EPA’s CompTox research is the Toxicity Forecaster (ToxCast™) [5], which is a multi-year effort launched in 2007 that uses automated chemical screening technologies, known as high-throughput screening assays, to expose living cells or isolated proteins to chemicals. The cells or proteins are then screened for changes in biological activity that may suggest potential toxic effects and eventually potential adverse health effects. These innovative methods have the potential to limit the number of required laboratory animal-based toxicity tests while quickly and efficiently screening large numbers of chemicals.

In the European Union, there is an ongoing long-term strategic initiative called the Safety Evaluation Ultimately Replacing Animal Testing [6], or SEURAT-1, to promote the intermediate steps that have to be taken before the final goal can be reached. SEURAT-1 will develop knowledge and technology building blocks required for the development of solutions to replace the current repeated dose systemic toxicity in vivo testing used for the assessment of human safety. The SEURAT-1 Research Initiative comprises six research projects, which will run for five years, starting on January 1, 2011. These projects promote close cooperation toward a common goal and combine the research efforts of over 70 European universities, public research institutes, and companies. Collaboration between these six research projects, dissemination of their results, cooperation with other international research teams, and continuous updating of research priorities will be facilitated by a related coordination and support action project called “COACH”.

A project in Japan called ARCH-Tox for the Future Chemicals Management Policy: Research and Development of in vitro and in vivo Assays for Internationally Leading Hazard Assessment and Test Methods is being supported by the Ministry of Economy, Trade and Industry (METI) [7]. This project aims to establish in vitro test methods for speedily and efficiently assessing the endpoint of 28-day repeated dose toxicities, hepatotoxicity, nephrotoxicity and neurotoxicity, and will promote close cooperation toward a common goal and combine the research efforts of six or more Japanese universities, public research institutes, and companies.

Also, the OECD continues its efforts to make better use of increased knowledge on the means by which chemicals induce adverse effects in humans and wildlife through Adverse Outcome Pathways (AOPs) [9]. Its efforts are based on knowledge of effective tools for identifying chemicals that need to be regulated. AOPs provide insight into how chemicals induce adverse effects through toxicity pathways and modes of action. Since 2012, the AOP Development Programme at the OECD has been pioneering the establishment of a comprehensive AOP framework for the effective use of mechanistic information in regulatory decision-making.

As a major step forward towards this goal, joint collaboration between the OECD, EPA, and the European Commission Joint Research Centre launched the Adverse Outcome Pathway Knowledge Base (AOP KB). This is a web-based platform which aims to bring together all knowledge on how chemicals can induce adverse effects, thereby providing a focal point for AOP development and dissemination. The first AOP KB module is the AOP Wiki: an interactive and virtual encyclopedia for AOP development, structured in accordance with the original OECD guidance document and template for developing and assessing adverse outcome pathways (Series No. 184, Series on Testing and Assessment) [10] and the more recent Handbook for AOP developers [11].

### “Introduction of ICH and recent topics related to safety” by Dr. Hiroshi Onodera, Pharmaceuticals and Medical Devices Agency

The development of novel medicines must focus on providing patients with safe and effective drugs on a global scale. The marketing of a drug necessitates the regulatory approval of the target country or region. However, since the documents and/or testing protocols required for registration may differ for each country/region, a considerable amount of time and energy might be required, which could result in a drug lag. From the viewpoint of animal welfare, it is important to avoid the unnecessary duplication of animal testing protocols. The International Conference on Harmonisation of Technical Requirements for Registration of Pharmaceuticals for Human Use (ICH) is a framework, which standardizes such requirements for registration, simultaneously ensuring the approval of safe, effective, and high-quality medicines.

The ICH combines the regulatory authorities and pharmaceutical industries in Europe, Japan, and the US. The ICH Steering Committee (SC) is the governing body that oversees the harmonization activities, while the Expert Working Group (EWG) is charged with the development of a harmonized guideline, based on a scientific consensus among experts. The topics are divided into four categories: quality, efficacy, safety, and multidisciplinary. The harmonization process is common across categories. The submission of a concept paper to the SC is followed by discussions, which are based on the agreement of the representatives from all regions. The harmonization process is advanced in a stepwise manner, from step 1 (consensus building) to step 5 (implementation). Currently, 10 guidelines for quality, 16 for efficacy, 10 for safety, and 7 guidelines for multidisciplinary fields have been harmonized (see references [12] and [13]). The M7 guideline (genotoxic impurities) has entered the fourth step of development in June 2014. Although this guideline was adopted by the EU on September 25, 2014 (step 5), it is yet to be validated in the US and Japan (as of Dec 2014). This time lapse in Japan could be because of the work related to translation and/or ensuring consistency with the domestic rules.

The major safety guideline currently under revision is the S1 guideline, which deals with rodent carcinogenicity studies for the development of human pharmaceuticals. The major objective of this review is to address the potential for elimination of the two-year rat bioassay, without compromising on patient safety. The existing S1 guideline necessitates the rodent two-year bioassay, based on the duration of exposure (>6 months, or repeatedly in an intermittent manner), a priori concern regarding the carcinogenic potential, and the clinical indication. On the contrary, no bioassay is required when there is sufficient evidence regarding carcinogenic concerns in humans (an indication of the genotoxic potential across species, long-term treatment using immune suppressants, etc.), or the technical difficulty of long-term administration. Instead, certain measures, such as the limitation of drug use or caution labeling, must be applied to reduce cancer risk in humans.

Current work on revision focuses on the prediction of negative result of bioassays based on the histopathology from chronic toxicology studies, and/or understanding the mode of action that does not evoke concerns in human. The ICH has initiated its first attempt of prospective data collection for analysis. The results of the prediction are to be compared with the outcomes of all bioassays. Although these investigations take a considerable amount of time, fruitful results would be expected.

### “Introduction of IWGT and recent topics” by Dr. Yoshifumi Uno, Mitsubishi Tanabe Pharma Corporation

The International Workshop on Genotoxicity Testing (IWGT) is a gathering of international experts from academia, government, and industry, focusing on the harmonization of genotoxicity testing methods and/or strategies for genotoxicity evaluation [14]. The IWGT is composed of working groups of topics, which are to be discussed internationally. Therefore, a chairperson, deputy chair, and rapporteur have been appointed for each topic, and the working group is organized with the experts [14].

The first IWGT (formerly IWGTP: the IWGT Procedure) was convened in Melbourne, Australia in 1993. At the time, the study protocols for genotoxicity testing had not been standardized globally; therefore, the study data obtained at a certain region could not be (often) accepted at other regions. Drs. T. Sofuni, M. Hayashi, and D. Kirkland played a central role in organizing this meeting, aiming to standardize genotoxicity testing. Although the International Conference on the Harmonization of Technical Requirements for the Registration of Pharmaceuticals for Human Use (ICH) has a similar objective, the IWGT encompasses other chemicals, in addition to the medical drugs reviewed by the ICH. Following the 4th IWGT (2005), it has been held every 4 years, in conjunction with the International Conference on Environmental Mutagens.

The working group members of the IWGT strive to advance focused discussions, and to derive recommendations based on a data-driven consensus [14]. All discussions are open to the audience; therefore, the audiences can also make a statement, in addition to the working group members. The consensus and/or the issues identified in the meeting is to be published in a scientific journal, e.g. Mutation Research [15, 16]. The most advantageous aspect of this meeting is that the discussions are based on the latest data/knowledge including unpublished information. Consequently, the outcomes of this discussion have been highly influential in the establishment and/or revision of the guidelines for genotoxicity testing, such OECD or ICH. In fact, the recommendations of the IWGT have often been used as the *de facto* guidelines for the testing of guidelines that remain to be established or revised as applicable.

The last IWGT, held in 2013 in Foz du Iguaçu, Brazil, focused on five subjects: comet assay, Pig-A assay, liver micronucleus assay, detection of germ cell mutagen, and quantitative genotoxicity risk assessment. In addition, next generation test strategies were also discussed. Many Japanese experts participated in all working groups, and contributed to achieving a consensus by providing critical data. Genotoxicity testing methods and/or the various strategies for evaluation are constantly evolving. Therefore, the importance of IWGT is growing, where expert scientists discuss the relevant topics based on the latest data, in regulatory science. The next IWGT has been scheduled in 2017 and is to be convened in Japan.

### “Introduction of Regulatory Science Working Group in JEMS and recent topics” by Dr. Masamitsu Honma, Division of Genetics & Mutagenesis, Biological Safety Research Center, NIHS

There is an increase in public concern regarding the safety of chemicals, such as food additives, pesticides, and industrial chemicals, used in foods and/or environment. The disaster at the Fukushima nuclear power plant induced the terror of developing cancer or hereditary diseases in the future among people. This rediscovered the fear of environmental carcinogens and the importance of ensuring the safety of chemicals in environment, among the people.

The Japanese Environmental Mutagen Society (JEMS) was established approximately 40 years ago, in order to adapt to the urgent need for the identification and evaluation of environmental mutagens, which emerged because of the drastic changes in environment during the period of high growth. Since then, JEMS has lead several research projects in this field, and has made an important contribution towards the identification and evaluation of mutagens. In addition, JEMS has also contributed to the establishment of the testing guidelines for genotoxicity evaluation, which is required for the approval of various regulations, for pharmaceuticals, pesticides, food additives, and industrial chemicals.

JEMS members must apply the results of their research or their experiences for the development of these guidelines, in order to assure the safety of the above chemicals based on sufficient scientific evidence, which would lead to a better society. This activity is considered as “regulatory science”.

The regulatory science working group of JEMS was established in 2013. The expert JEMS members volunteering as a part of this group provide their comments or data on the drafting or revision of the testing guidelines (OECD, ICH, etc.). The initial 40 members consisted of 5 experts from universities/academia, 11 from public institutes, 9 from CROs, and 15 from the pharmaceutical/chemical industries. With the revision or abolition of the existing OECD genotoxicity testing guidelines, the members of the working group often provide comments to format these to the current context [17].

A new ICH guideline, M7, has been recently established to assess and manage the genotoxic impurities in pharmaceuticals [18]. This guideline is believed to define new approaches to facilitate a paradigm shift in genotoxicity evaluation. One such approach is the application of the Thresholds of Toxicological Concern (TTC), in order to determine the need for further risk assessment of impurities with low-level exposures. The acceptable limit of mutagenic impurity is 1.5 μg per person per day, which is considered a negligible risk (theoretical excess cancer risk of <1 in 100,000, over a lifetime of exposure). This criterion is derived from risk assessment, but not from hazard identification. Another approach is the acceptance of an *in silico* approach to address the mutagenic concern. The (quantitative) structure-activity relationship ((Q)SAR) is a powerful *in silico* technique used to prioritize or screen chemicals during the development of pharmaceuticals [19]. ICH-M7, however, considers the (Q)SAR to be a regulatory test assisting in decision making during hazard assessment. The absence of structural alerts from two complementary (Q)SAR methodologies (expert rule-based and statistical) would be sufficient to conclude that the impurity is of no mutagenic concern. Therefore, no further testing would be recommended. These provisions are expected to promote the trend from hazard identification to risk assessment, and from ordinary testing to intelligent system analysis.

In conclusion, the standard of regulatory science has been altered to adapt to the social demand of the age. There is a lot of discussion regarding the meaning of and need for regulatory science. However, according to his biased opinion, regulatory science can be considered as a “moral science”, which provides the scientific basis for conforming to the current societal norms.

### “Problems assessing food’s effect on human health” by Dr. Naoko Koizumi, former chairperson, Food Safety Commission of Japan

To assure food safety in Japan, the functions of risk assessment and risk management are separate and conducted by the Food Safety Commission of Japan (FSCJ) and other regulatory authorities. This may not be the best approach; there are pros and cons. One of the advantages is that hazard assessment is conducted in a scientific and neutral manner. Nevertheless, there are still various problems and/or challenges. From the viewpoint of public health, she has been involved in the assessment of food’s effect on human health. She illustrated the problems found in the following areas relevant to risk assessment: 1) in the assessment of food’s effect on human health, there are problems in each step of Plan-Do-Check-Act cycle; 2) the current operation of the FSCJ; 3) challenges in the future.

Finally she pointed out that there are many problems and challenges for improvement of these areas. She expects her talk will inspire the people involved to increase their awareness of these issues and address these concerns.

### “Challenges for regulatory science in Japan” by Dr. Makoto Hayashi, Public Interest Incorporated Foundation BioSafety Research Center

“It is very easy to over- or underestimate risk, but very hard to make a rational assessment of risk.” This comment was made by Professor Torahiko Terada, a famous novelist and scientist in physics and translated into English by Professor Sohei Kondo. Regulatory science should promote a rational assessment of risk. Although there are many challenges for regulatory science, the following are considered focus points.

1. Expand understanding of regulatory science

The first priority is to expand understanding of regulatory science among laypeople. He thinks understanding the following two ideas are particularly important for rational assessment: i) Hazard vs. Risk. Many people do not see the difference between these two words. For example, tobacco is hazardous material. However, if you do not smoke (i.e., no exposure), there is no risk; ii) Zero risk. We need to understand that any activity/substance possesses risk. Even oxygen, which is necessary for our life, poses a risk. Therefore, “zero risk” is not possible in reality and pursing it will result in increasing other risks.

2) Training of regulatory scientists

Risk can be considered a function of multiple factors, including hazard, exposure, target population, and more. There are many technical hurdles to overcome to draw conclusions. In relation to genotoxicity, considering the threshold is an important and difficult issue. Thus, risk assessment comprises various steps, and the assessor needs to review a large volume of information. However, there is no systematic training for regulatory scientists. Education of experts capable of risk assessment is urgent.

3) Risk communication

Communicating the outcome of risk assessment is also a key challenge for us. Relevant stakeholders are diverse and include consumers, producers, risk assessors, risk managers, and others. It is difficult to find an effective way to communicate with all the stakeholders. Nevertheless, communication will determine the success or failure of the risk assessment. We need to explore better approaches for communicating the risk.

4) New initiatives

New projects or concepts, such as Tox21, AOP, RISK21, and Risk/Benefit analysis, have been initiated in the field of regulatory science. These can offer breakthrough approaches to overcome the challenges described above. We need to watch the developments of these concepts closely or be involved in the development of such activities.

### “A key issue for risk assessment: threshold on carcinogenesis” by Dr. Shoji Fukushima, Japan Bioassay Research Center, Japan Industrial Safety & Health Association

In order to assess the risk associated with a carcinogen, the carcinogenicity of the target chemical must first be assessed. Based on an analysis of the exposure status, the possibility of tumor occurrence (risk assessment for carcinogenesis) can be assessed.

In general, a chemical elicits a biological response in a dose dependent manner. The upper-limit of the range in which no response is observed is called the threshold level. Therefore, in general toxicity analysis, it is critical to determine the threshold of a chemical to ensure safety. However, this raises the question regarding the methods for assessing carcinogenic risk. Thus far, no threshold level has been set for the evaluation of the safety of a carcinogen, especially that of a genotoxic carcinogen. However, this premise has not been scientifically proven.

Therefore, the carcinogenicity of various environmental carcinogens was analyzed in rats at low dose levels, based on the “weight of evidence” [20]. Most genotoxic carcinogens or their metabolites covalently bind to DNA, forming adducts that lead to mutation. The initiated cells (mutated cells) proliferate to form pre-neoplastic lesions, such as GST-P positive foci, which further develop into tumor. This sequence of events is generally observed during chemical carcinogenesis. For example, 2-amino-3,8 dimethylimidazo[4,5-f]quinoxaline (MeIQx), which is found in cooked fish or meat, is known to provide a positive response to the Ames test and to induce tumors in the rat liver. Rats administered MeIQx at a wide range of doses (0.001–100 ppm) developed a MeIQx-DNA adduct (8-hydroxy-2′-deoxyguanosine (8-OHdG)) as an oxidative stress marker, and the GST-P positive foci, in an increasing order of dosage, in the liver. These markers did not express any detectable changes at the lowest dosage. This finding was indicative of the existence of a no-effect level.

N-nitrosodiethylamine (DEN) is known to form in the stomach because of the reaction between the ingested secondary amines and nitrites. DEN also provides a positive response to the Ames test and induces tumors in the rat liver. Big Blue rats administered with DEN for 16 weeks at a wide range of doses (0.0001–1 ppm) developed GST-P positive foci, as well as an elevated mutation frequency in the *lacI* gene, in an increasing order of dosage, in the liver.

Accordingly, Dr. Fukushima and his colleagues demonstrated that heterocyclic amines and/or nitrosamines, which gave a positive response in the Ames test, had a practical threshold level for carcinogenesis in rats.

Furthermore, an approach has been initiated by academia to evaluate the genotoxicity from a quantitative, rather than a traditional qualitative viewpoint [21, 22]. The development of such an approach would lead to innovations in the risk assessment of genotoxic carcinogens.

### “Comparison of Japanese and overseas risk assessment committees” by Dr. Hiroshi Yamasaki, former Kwansei Gakuin University

In an increasingly globalized world, it is important to establish a consensus on the international risk assessment processes. He worked for the International Agency on Research on Cancer (IARC), and was involved in monograph meetings on the evaluation of carcinogens affecting humans. Further, on a request from the USA, he also joined the committee of the National Toxicology Program (NTP) for the evaluation of carcinogens as a scientific expert.

After returning to Japan, he had the opportunity to watch the discussions in the Food Safety Commission of Japan (FSCJ). Based on his experience, he compared the approaches of the FSCJ and similar organizations overseas (mainly the USA, Europe, and Australia) to identify preferred organizational structures for risk assessment.

The IARC and NTP are not organizations that address risk assessment; these organizations are involved in hazard identification. However, risk assessment begins with the identification and evaluation of hazards. Since scientific data necessary for risk assessment are also gathered and reviewed, the process of hazard evaluation is very important. While IARC and NTP do not address “potential” carcinogens, instead focusing on those identified by the “weight of scientific evidence,” risk assessment needs to evaluate the “potential” of the carcinogen through the incorporation of various information, including exposure to the hazardous substance and the potential genetic susceptibility of the target population. In the USA, NTP conducts “hazard evaluation,” while the Environmental Protection Agency (EPA) and the Food and Drug Administration (FDA) conduct “risk assessments.” The FSCJ, founded ten years ago to secure food safety in Japan, conducts both hazard evaluation and risk assessment. In Europe, food risk assessment is conducted by the British Food Standards Agency (FSA) in England, the Federal Institute for Risk Assessment (BfR) in Germany, and the European Food Safety Authority (EFSA) for the European Union (EU). In Australia and New Zealand, the Food Standards Australia New Zealand (FSANZ) conducts risk assessment for food. From his survey on these organizations, the differences between the FSCJ and the overseas organizations include the following.

1. Evaluation period

The monograph meetings for the evaluation of potential human carcinogens at IARC are usually held for 7–8 days and reach a conclusion within that period. Generally, the evaluation meetings held at the NTP complete discussion and draw a conclusion within 2–3 days. The EFSA sets a period of 3–9 weeks from the start of evaluation to a conclusion and they adhere to this schedule. On the other hand, the FSCJ has no timeframe for reaching a conclusion; therefore, the discussion occasionally continues for a long period of time. In the case he watched, no conclusion has been reached on a potential carcinogen in the more than 10 years since the first discussion.

2) Data used for evaluation

As a rule, the IARC and NTP use only published scientific data for evaluation, and the EFSA focuses on published data. Meanwhile, the FSCJ, although they declare an emphasis on published data, often use unpublished data submitted by scientists commissioned by the government for evaluations.

3) Cooperation with industries

Most of the above committees, except the FSCJ, select the members involved in the discussion from industry scientists or consultants. In general, an industry representative is knowledgeable about the issue and has data to add to the discussion. In the IARC and/or NTP, a few scientists from the industry participate in the process of evaluation; however, they do not have the authority to make judgments. Based on his experience, he believes including representatives from the industry is necessary for a fair evaluation.

Based on the differences identified above, he proposes that the FSCJ take the following actions to improve their risk assessment committees. First, the FSCJ needs to separate “solution-focused” from “problem-focused” discussions to allow conclusions using publically available scientific data to be reached by a pre-established target deadline. Second, the FSCJ needs to cooperate with all stakeholders, especially those from industry. Discussions that include other stakeholders will result in a fair and speedy evaluation.

### Panel discussion

Panel members included the following five lecturers of the symposium: Dr. Naoko Koizumi (NK), Dr. Makoto Hayashi (MH), Dr. Shoji Fukushima (SF), Dr. Jun Kanno (JK), and Dr. Hiroshi Yamasaki (HY). Moderator was Toshio Kasamatsu (TK). Details of the panel discussion are described in the Additional file 1.

## Conclusion

As many as 154 people participated in the symposium, based on the number of leaflets distributed. The participants were asked to answer a questionnaire; 57 answers were received in total from JEMS members (19; 33 %), and non-members (30; 53 %), and people who left no records (8; 14 %). This indicated that over half the participants were non-JEMS members. The feedback received included favorable (“Symposium was very interesting”, “It was very helpful to obtain an overview of regulatory science”, and “It was great to understand the difference between risk assessment and risk management”), as well unfavorable/critical (“Symposium did not fully focus on regulatory science”, “There were few talks on the idea or the standard of regulatory science”, and “Not showing vision for the specific measures adopted by JEMS towards the development of a young successor, including the potential of JEMS itself, or what JEMS can accomplish by approaching the government, etc.”) comments.

An analysis of the symposium, based on the comments presented above, revealed the following points; the lectures presented the current achievements of JEMS, as well as the challenges that remain to be addressed; the important contribution made by JEMS towards the establishment of regulatory science was well recognized; on the other hand, basic framework regarding the assessment of genotoxicity has remained unchanged over the past decades. Regarding the final point, the Ames test, which was developed approximately 40 years ago, remains to be the golden standard in genotoxicity testing, despite the development and proposal of various new testing strategies for regulatory applications. Upon obtaining a positive result in the Ames test, the tested chemical would be treated as a mutagen. Alleged genotoxicity and/or carcinogenicity concerns have led to the setting of a high standard for regulatory decision making, regarding the safety of chemicals. Nevertheless, TTC, a novel concept explained by Dr. Honma, and the genotoxicity threshold model proposed by Dr. Fukushima et al., might provide the vital clue towards establishing novel frameworks for regulatory science.

The panel discussion also revealed important challenges to be overcome for the development of regulatory science in Japan. These include: 1) the lack of an established approach for the evaluation of safety of entire food, 2) the urgent need for development and appointment of young successors, and 3) the involvement of industry representative in evaluation, etc. There is no simple solution detailing the contribution of JEMS towards addressing these challenges, However, JEMS should be able to attractively present young scientists with challenges worth working on, as well as support their ideas for the establishment of new standards for regulatory science.

As the coordinators of the symposium, we are also attempting to contrive better ways to communicate the concept of regulatory science. Nevertheless, we believe it was a great opportunity to elicit interest in many members of the audience regarding regulatory science. As explained in the Introduction, the major objective of this symposium was to rediscover the role played by JEMS in the development and propagation of regulatory science, and to provide the people (including JEMS members) with an opportunity to discuss the meaning of regulatory science, and strategies for its development in Japan. Although there are several concepts clarifying the above issues, it would be helpful if all members of the audience, as well as the readers of this article, would attempt to clarify these points.

Based on a request in the questionnaire, the presentation materials of the lecturers have been up-loaded, with due permission, on the JEMS homepage <http://www.j-ems.org/symposium/2014symposium.html>. We hope that this would help understand regulatory science.

## Additional file

Additional file 1:
**Panel discussion (JEMS symposium 2014).**
