# Supplementary material for: Regulatory science - JEMS symposium in 2014
Source: Genes Environ. 2015 Jul 30;37:12. doi: 10.1186/s41021-015-0012-x (PMC4917975; doi:10.1186/s41021-015-0012-x)
Supplement: Additional file 1: — Panel discussion (JEMS symposium 2014). [file 41021_2015_12_MOESM1_ESM.docx]

**Panel Discussion**

Panel members included the following five lecturers at the symposium: Dr. Naoko Koizumi (NK), Dr. Makoto Hayashi (MH), Dr. Shoji Fukushima (SF), Dr. Jun Kanno (JK), and Dr. Hiroshi Yamasaki (HY). Moderator was Toshio Kasamatsu (TK).

The discussion began with questions posted by the audience.

Q1. Except for medical drugs, I feel the development of regulatory science (RS) dealing with food and/or genetically modified organisms (GMO) is rather slow. Although there are many novel foods or GMOs, we cannot expand the use of these products due to strict regulation and/or evaluation.

Q2. Secondly, I would like to know what regulatory authorities think about RS.

NK: Regarding Q1, I think that, conclusively, risk communication is key to addressing the issue. Our survey revealed that consumers were very afraid of these products, even though we explained that their safety had been carefully evaluated. For example, there is no need to display “GMO” when a product is made up of less than 5% GMO. I wonder whether such ambiguous standards will lead to consumer concerns.

MH: Regarding Q2, for example, Chemical Control Law has been working to move from hazard identification to risk assessment. They make a point to conduct exposure assessments. Regardless of whether “RS” is used or not, regulatory authorities are conscious of risk management.

SF: Regarding Q2, currently my concern lies with increasing the number of young scientists who study RS. When I was a member of the Food Safety Commission of Japan (FSCJ), I searched for a potential candidate to be my successor, but the possibilities were very limited. While I am also a referee for research funding at the Ministry of Health, Labour and Welfare (MHLW), the MHLW has cut funding to the grant program for young scientists; they can hardly make a living. I am worried about if this is right for Japan. I would like to ask you (the audience) to raise your voice against the current situation.

JK: Returning to the topic of GMO, I feel the key is communication. I usually do not talk about “safe and assured,” but I dare to use these words today. Once a seed of anxiety, which is the opposite of assured, is sown, it grows. I feel that only communication cannot solve the issue as communication reminds me of a one-way argument. Therefore, I proposed a “Round Table” as a method to allow every stakeholder to participate. I learned that, in England, after a failure in handling bovine spongiform encephalitis (BSE) issue, the government organized a crisis management group that is continuing to do well (e.g. there was no panic over the Fukushima disaster). There is no reason that we cannot do the same. We can draw on their experience. Regarding Q2, the reason why young scientists are not in this field is that there is no faculty or place for studying and doing toxicology. If no roadmap for a career is available, it will become difficult to find successors. Regarding the question about how regulatory authorities consider RS, it is not easy to talk about here, yet I have certain reservations about it.

HY: From the viewpoint of outside Japan, as Dr. Hayashi talked about with regard to rational risk assessment, I feel Japanese people are too afraid of risk. The mass media also seem to report based on “zero risk.” I think the JEMS is well balanced between academia and industry, and this is unusual for Japan. I expect that the JEMS will take a leadership role in changing the current circumstances.

Q3. I would like to know your opinion of the evaluation of unidentified toxic substances found in food.

NK: The FSCJ evaluates food based on scientific information collected. As the FSCJ cannot carry out its own research, they must ask relevant ministries and/or agencies to provide information for evaluation. If a substance is unidentified, risk assessment cannot be carried out. The FSCJ will send the case back to the applicant.

MH: Although I am not the right person to answer this question, unidentified substances cannot be evaluated by a group such as the FSCJ. When we want to evaluate, we evaluate the entire food. But, I think it is very difficult to evaluate unidentified substances because there is no established approach to evaluating the food containing said substances.

SF: As analytical technology has dramatically improved, it is impossible to evaluate all substances detected. The idea of Threshold of Toxicological Concern (TTC) needs to be applied for evaluation seriously.

JK: It may be important to know why you want to evaluate unidentified substances. If there is no reason, there is no doubt.

HY: I have the same opinion as Dr. Kanno. What is the purpose for evaluation? Although I do not know about the FSCJ, the International Agency for Research on Cancer (IARC) has two conditions for evaluation; (1) human exposure to the substance, and (2) the possibility of carcinogenesis has been suggested. It would be terrible if a substance were evaluated when these conditions were not fulfilled.

NK: In the FSCJ, first, there is discussion about evaluating this hazard. For unidentified substances, risk management authorities gather and examine relevant information to determine if discussion is necessary. When their answer is ready, they consult the FSCJ.

Q4. There was talk of proposing the involvement of industry representatives in evaluation. In the NTP, it is clearly reported who votes pro and con. I heard that for the TSCA registration in the US, the person in charge of the company that submitted the notice must answer all questions raised by the authorities. For the Chemical Control Law registration, I wonder if it is better to give an industry representative who is involved with the substance the opportunity for discussion even though he/she does not evaluate it.

MH: Regarding the Chemical Control Law registration, although it is not an evaluation, the industry can exchange opinions with the authorities through a consultation desk. The FSCJ also decided to exchange opinions with the pesticides industry with limitations.

NK: There has been discussion that since it is time consuming and troublesome to obtain necessary information one month later and then deliberate, it is better to suggest asking a company representative to stand by when there are questions needing answers. As the law concerning the FSCJ states “neutral, fair, and scientific” review, the secretariat adheres to it. Meanwhile, the FSCJ discloses all processes. You can check the proceedings immediately and express your opinion as a public comment when an evaluation report is made. In this respect, I think this is an advantage of the FSCJ compared to overseas organizations, such as the European Food Safety Authority (EFSA), in which the evaluation process is not disclosed.

MH: Let me add an explanation to my previous comment. Exchanging opinions with the industry is limited to the pesticide section for new pesticide registrations. It is expected be closed discussion.

SF: I pass on this question. Let me talk about something different. Initially, as an expert panel member, I was uncomfortable with the disclosure of the proceedings. But, once I got used to it, I did not care anymore.

JK: When I participated in overseas meetings, such as the OECD evaluations, I had chances to discuss the data with industry representatives and felt comfortable with this. Regarding the reasons that such circumstances have not been used in Japan, I wonder if a change of secretariat every two years results in a lack of continuity and may be a problem. Because a person cannot be assessed accurately, there is too much worry about neutrality. A generous environment for maintaining neutrality could not be present unless an officer keeps the same position for about 20 years, as in the US.

HY: Related to this conversation, many overseas evaluation committees have laboratories doing research. I think the National Institute of Health Science (NIHS) should take the role of the secretariat for the FSCJ. Involvement of experts who understand the science in the management for the long term would enable fairness and durability. Although disclosure of all processes in the FSCJ is good, it is most meaningful when all stakeholders participate. It is meaningless if only the government and academia participate.

Q5. There was a lecture by Dr. Yamasaki that mentioned overseas evaluation committees setting a timeline for evaluation. When we need to make a decision, it is almost impossible to have all the required information. What is a reason for there being no pressure to set a timeline or make a conclusion by the set time in Japan?

HY: Members need to be aware that the organization is not meant for research, but rather for evaluation. The participation of an industry representative is also important in this context. I think it will generate a better atmosphere for reaching conclusions at some point. Meanwhile, it does not help that the time for judgments differs between hazard evaluations and risk assessments because the assessor’s mental pressure is different.

JK: I was once told that the head of an evaluation organization took responsibility for the evaluation result. Although it is generous, my responsibility is ambiguous, so I feel uneasy in taking responsibility for an evaluation.

HY: It is the same in overseas organizations. In the IARC, as the director is requested to act, the director takes the ultimate responsibility.

MH: There is no expert working full-time in the secretariat. I think it is one of reasons why evaluations tend to be prolonged. The assessors participate voluntarily, so they may have only a limited time to spend on evaluations. Although this might only be true in the pesticide section, the FSCJ suggests that a decision should be made within one year and I think they keep the time-clock.

NK: I think it is critical to draw conclusions as early as possible for the protection of public health. I learned that in the EFSA, the senior organization determines how many days are necessary for a hazard evaluation before starting the evaluation. I wonder if we can take a similar approach. Define separate roles for the parent committee and expert panel, specifically, and have the parent committee determines the period of evaluation before the expert panel deliberates. Once a change of secretariat occurs, the evaluation goes back to the beginning. A technical advisor who prepares an original evaluation document has good skills and long experience, but limited authority. If mangers from the MHLW or the Ministry of Agriculture, Forestry, and Fishery (MAFF) are unable to choose information, they send all the information to the expert panel. At that point, the expert panel members may feel many discussions are needed and the evaluation period should be prolonged due to the amount of material.

Q6. Although it seems there is less interest in reducing animal experimentation in Japan, many efforts for evaluations without animal testing have been made overseas. In a case that we need to evaluate, even though there is no applicant, I think we may need to refer to the overseas evaluation results. Could you please give your opinion on a possible occasion when there is no animal data available for evaluation?

NK: In principle, the MHLW and/or MAFF discuss a case with the industry first and then consult the FSCJ. The FSCJ does not directly review data from the industry and determine if there is a risk. Except for consultations, the FSCJ conducts self-evaluations by gathering information from the literature.

MH: Although this may only be the case in the pesticide area, when the positive list system was introduced the FSCJ evaluated documents from overseas about a chemical when other data were not available.

SF: I see such movement, certainly. Although it is case by case, my position is that in vivo data is necessary for evaluations.

JK: The idea of 3R has been incorporated at research sites. It would be appreciated if you understood that we are working to establish a framework to adapt to these cases. However, it would not be easy to utilize *in vitro* or *in silico* approaches to evaluate cases that could not be elucidated by *in vivo* analyses.

HY: The evaluation process is heavily dependent on the science at the time. When we understand the mode of action, we can use it for evaluation. I think that, although animal experiments would continue, the rate of evaluations relying on them would decrease.

TK: It is a time to close the panel discussion. Are there any further comments?

SF: What happens to today’s output? How is it conveyed to the regulatory side? Another comment is that I wonder if the word “trust” is better than “safe and assured.” A baby feels assured because he/she trusts the mother. It is not true that he/she trusts the mother because of assured. Emphasizing “assured” would result in pursuing this notion of “zero risk.”

TK: Regarding output, a special issue of *Genes & Environment*, a journal of the JEMS, is scheduled to publish today’s discussions. I plan to compile a manuscript on the content of today's panel discussion and submit this to the journal with the agreement of all panelists. I believe this will help communicate today’s discussion to public. Furthermore, many of the lecturers and members of the audience are influential in regulatory agencies and, given the opportunity, I would like to ask you to communicate today’s discussions.
